# *EVI2B* Is a New Prognostic Biomarker in Metastatic Melanoma with IFNgamma Associated Immune Infiltration

**DOI:** 10.3390/cancers13164110

**Published:** 2021-08-15

**Authors:** Satoru Yonekura, Kosuke Ueda

**Affiliations:** 1 Gustave Roussy Cancer Campus (GRCC), 94800 Villejuif, France; 2 Department of Urology, Kurume University School of Medicine, Kurume 830-0011, Japan; ueda_kousuke@med.kurume-u.ac.jp

**Keywords:** melanoma, biomarker, *EVI2B*, microenvironment, IFN-γ, tumor-infiltrating

## Abstract

**Simple Summary:**

Ecotropic viral integration site 2B (*EVI2B*) is a protein-coding gene known as a lymphocyte-specific marker in peripheral blood. However, the prognostic value of *EVI2B* expression in metastatic melanoma tissue and its detailed profile of tumor-infiltrating lymphocytes are still unclear. In publicly available datasets, we found that increased *EVI2B* was significantly associated with longer prognoses such as overall survival and disease-specific survival. The *EVI2B*-high melanoma tissue had a favorable distribution/clustering pattern of infiltrating lymphocytes with increased CD8+ T cells over regulatory T cells. Moreover, *EVI2B* expression correlated with multiple immunomodulatory genes including IFN-γ signature genes. In conclusion, *EVI2B* is a prognostic biomarker with IFN-γ associated immune infiltration in metastatic melanoma.

**Abstract:**

Background: To assess the prognostic role and the antitumor immunological relevance of ecotropic viral integration site 2B (EVI2B) in metastatic melanoma. Methods: In this study, we integrated clinical data, mRNA expression data, and the distribution and fraction of tumor infiltrating lymphocytes (TILs) using The Cancer Genome Atlas (TCGA) and Gene Expression Omnibus (GEO) datasets (GSE65904 and GSE19234). Results: The univariate and multivariate analyses showed that higher gene expression of *EVI2B* was significantly associated with longer prognoses. The *EVI2B*-high melanoma tissue had favorable histological parameters such as a brisk global distribution pattern and clustering structure of TILs (i.e., Banfield and Raftery index) with enriched CD8+ T cells over regulatory T cells and increased cytotoxicity scores. In addition, *EVI2B* expression positively correlated with IFN-γ signature genes (*CXCL10*, *CXCL9*, *HLA-DRA*, *IDO1*, *IFNG*, and *STAT1*) and other various immunomodulatory genes. Conclusion: *EVI2B* is a novel prognostic biomarker with IFN-γ associated immune infiltration in metastatic melanoma.

## 1. Introduction

Cutaneous melanoma is a malignant tumor originating from melanocytes [1]. Although the incidence rate of melanoma is lower than other skin cancers, it is responsible for most metastatic skin cancer–related deaths [2,3]. Melanoma is an immunogenic tumor with multiple types of tumor-infiltrating lymphocytes (TILs) in the tumor microenvironment (TME) [4]. The distribution and density of TILs are predictors of survival outcomes [5]. Moreover, the TME profile is closely associated with the therapeutic success of immune checkpoint blockade [6]. Therefore, analyzing the TME of melanoma may help better understand the immunological interactions between the tumor and the host cells and how such interactions influence the clinical outcomes.

Ecotropic viral integration site 2B (*EVI2B*), also known as CD361 [7], was first reported to be a common viral integration site in retrovirally-induced murine leukemia [8]. Zjablovskaja et al. showed *EVI2B* was necessary for granulocytic differentiation and functionality of hematopoietic progenitors [9]. *EVI2B* is one of the lymphocyte-specific attractor metagenes in multiple tumor samples such as breast cancer, colorectal adenocarcinoma, and ovarian cancer [10] and predictive of progression in colorectal cancer [11]. Since melanoma can be enriched with infiltrating lymphocytes, *EVI2B* could have a potential association. However, no study has previously investigated *EVI2B* as a potential biomarker in metastatic melanoma.

In this study, we investigated the prognostic value of *EVI2B* gene expressions using the metastatic melanoma datasets from The Cancer Genome Atlas (TCGA) and Gene Expression Omnibus (GEO) datasets. We characterized the TME using spatial distribution pattern (brisk vs. non-brisk) [12], statistical indices of clustering pattern [12,13,14,15,16], and estimated fractions of TILs [17]. Finally, our evaluation showed that *EVI2B* was associated with gene signatures that were associated with a better response to immune checkpoint blockades such as anti-PD1 or anti-CTLA4 antibody therapies [18,19]. 

## 2. Materials and Methods

### 2.1. Survival Analysis with EVI2B mRNA Level in the Public Database 

Clinical data of patients with metastatic melanoma were obtained from cBioPortal (TCGA PanCancer Atlas, https://www.cbioportal.org/, accessed on 20 March 2020) [20,21]. OSskcm, an online tool for survival analysis with transcriptome information [22], was used to obtain the clinical data of GSE65904 [23] and GSE19234 [24], which both mostly consist of metastatic melanoma with gene expression data in tumor tissue. We classified the patients with upper 25% expression of *EVI2B* as *EVI2B* high and the remaining patients as *EVI2B* low in all the following analyses. For the visualization of the overall survival (OS) and disease-specific survival (DSS), we used Kaplan–Meier plotting. The multivariate Cox regression analysis was performed to assess the prognostic impact of *EVI2B* gene expression as a continuous variable with the other parameters such as age at diagnosis, sex (male vs. female), disease stage at initial diagnosis, and tumor purity on tumor immune estimation resource (TIMER, https://cistrome.shinyapps.io/timer/, accessed on 6 December 2020, TIMER2.0, http://timer.comp-genomics.org/, accessed on 6 December 2020) [25,26].

### 2.2. Estimated Fractions of TILs

The TIL fraction data estimated by the quanTIseq [17], TIMER [25,26,27], and Microenvironment Cell Populations counter (MCP-counter) [28] were downloaded from TIMER2.0 (http://timer.comp-genomics.org/, accessed on 6 December 2020) [26]. In addition, CIBERSORTx was used to estimate the TIL population using LM22 as signature with 100 permutations [29]. The heatmap of TIL fractions estimated by quanTIseq [17] or CIBERSORTx [29] combined with clinical information was generated by R package ”ComplexHeatmap”. Each fraction of TILs was converted to a z-score. We used non-hierarchical clustering for characterizing the TILs. The calculating method of the distance for rows and columns was ”Canberra”. The clustering method for rows and columns was ”ward.D” or ”ward.D2”. Multiple t-tests with false discovery approach [30] were used to compare the estimated TIL fractions between *EVI2B* high vs. *EVI2B* low patients.

### 2.3. Patial Pattern of TIL Analysis

The spatial TIL distribution patterns were downloaded from the Genomic Data Commons (GDC) data portal (https://gdc.cancer.gov/about-data/publications/tilmap, accessed on 20 March 2020) [12]. Samples with a pattern label of ”indeterminate” were excluded from the analysis. The distribution pattern of TILs was compared between *EVI2B* high and *EVI2B* low patients by Fisher’s exact test. We downloaded the computed indices data of the clustering pattern of TILs (determinant ratio [13], C [14], Banfield and Raftery [15], and Ball and Hall [16]) from the GDC data portal (https://gdc.cancer.gov/about-data/publications/tilmap, accessed on 20 March 2020) [12].

### 2.4. Correlation with Sets of Immunomodulatory Genes

The Spearman correlation of *EVI2B* mRNA level with IFN-γ signature gene levels (*IDO1*, *CXCL10*, *CXCL9*, *HLA-DRA*, *STAT1*, and *IFNG*) [18] and anti-CTLA-4 resistance-associated MAGE-A (CRMA) genes (*CSAG1*, *CSAG2*, *CSAG3*, *MAGEA2*, *MAGEA3*, *MAGEA6*, and *MAGEA12*) [19] were investigated using GEPIA2 [31]. We excluded *MAGEA2B* because the expression data were not available on GEPIA2 for metastatic melanoma. The correlation of *EVI2B* with two sets of genes (immunostimulators and immunoinhibitors [32]) were also investigated.

### 2.5. Statistical Analysis

Spearman’s rho was used for correlation analyses. Log-rank test was performed by the OSskcm [22]. For graphs and statistical analyses (multiple *t*-tests with false discovery rate (FDR) approach, Mann–Whitney test, and Fisher’s exact test), we used the R freeware (http://www.r-project.org, accessed on 14 December 2020) and GraphPad Prism (v. 8.3.0) software (GraphPad Software Inc., San Diego, City, CA, USA). All *p*-values were two-sided, and a *p*-value and an FDR (Q value) of ≤0.05 were considered statistically significant.

## 3. Results

### 3.1. Prognostic Impact of EVI2B Gene Expression in Metastatic Melanoma

We investigated the survival data from TCGA in metastatic melanoma patients with *EVI2B* gene expression. The third quartile of *EVI2B* mRNA level was chosen as a cutoff to stratify patients as “*EVI2B* high” and “*EVI2B* low” because the distribution of the *EVI2B* mRNA levels was positively skewed (Figure S1). The basic characteristics of the evaluated patients are shown in Table 1. No difference in age, sex, race, and TNM stage was observed between *EVI2B* high and *EVI2B* low patient groups.

**Figure 1:**
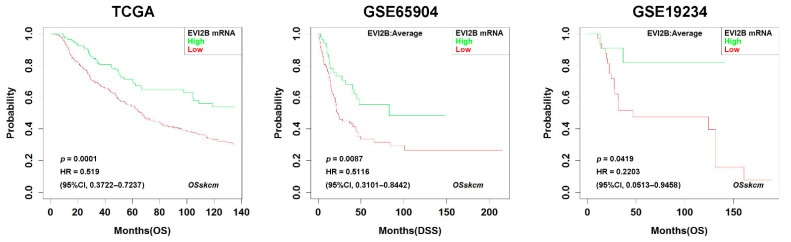
Kaplan–Meier survival curves in *EVI2B* high vs. *EVI2B* low patients with metastatic melanoma in TCGA, GSE65904, and GSE19234. The outcomes are overall survival (OS) (**left** and **right panels**) and disease-free survival (DSS) (**middle panel**).

Patients with higher *EVI2B* gene expressions had longer OS (hazard ratio (HR) (95% confidence interval (CI)) 0.519 (0.372–0.724), *p* = 0.0001, Figure 1 left panel). Similarly, in the other cohorts (GSE65904 [23] and GSE19234 [24]), the *EVI2B* gene expression was a favorable factor on DSS and OS (HR (95% CI) 0.512 (0.310–0.844), *p* = 0.009 and HR (95% CI) 0.220 (0.051–0.946), *p* = 0.042, respectively, Figure 1 middle and right panels). We found a similar prognostic value of *EVI2B* with a 50% cut-off value in the TCGA, GSE65904 [23], and GSE19234 [24] datasets (Figure S2).

The multivariate Cox regression analysis also revealed that a higher *EVI2B* gene expression as a continuous parameter was significantly associated with longer OS (HR (95% CI) 0.716 (0.610–0.840), *p* < 0.0001, Table 2) in the TCGA cohort. The dichotomized *EVI2B* mRNA level (high vs low) was an independent prognostic factor in the multivariate Cox regression analysis as well (Table S1).

### 3.2. Spatial Pattern of Infiltrating Immune Cells by EVI2B Gene Expression

**Figure 2:**
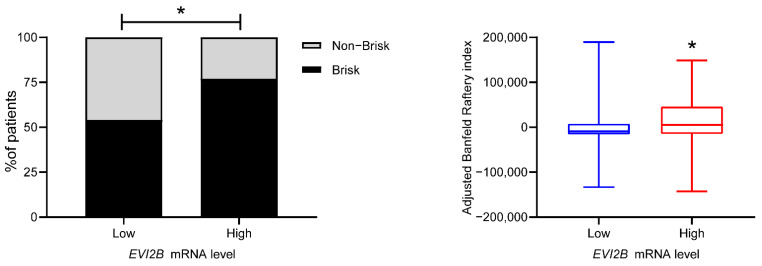
Spatial pattern of TILs in *EVI2B* high vs. *EVI2B* low patients: (**left**) Stacked bar plot of the distribution patterns of TILs (brisk or non-brisk), * *p* < 0.05 by Fisher’s exact test; (**right**) adjusted Banfield and Raftery index of TILs clustering patterns, *: *p* < 0.05 by Mann–Whitney test.

The spatial structure of infiltrating lymphocytes in tumor tissue such as global distribution and clustering patterns of TILs is vital to predicting patient prognosis [12]. We compared the distribution of TILs described in a previous study [12] between the *EVI2B* high vs. *EVI2B* low patient groups. Seven patients with the indeterminate pattern were excluded. In the *EVI2B* high patient group, 77.3% (58/75) had a brisk pattern (band-like and brisk diffuse), whereas 22.7% (17/75) had a non-brisk pattern (non-brisk multifocal, non-brisk focal, and none). In the *EVI2B* low patient group, almost half of the included patients had a brisk pattern (54.3%, 119/219). The distribution pattern between the *EVI2B* high and EVI2B low patient group was significantly different (*p* = 0.0002, Fisher’s exact test, Figure 2 left panel).

Computed clustering patterns of TILs such as determinant ratio [13], C [14], Banfield and Raftery [15], and Ball and Hall [16] are associated with prognosis in cancer patients [12]. A higher Banfield and Raftery index was significantly associated with better prognosis in TCGA melanoma patients [12]. In our study, the Banfield and Raftery index was significantly higher in the *EVI2B* high patients (Figure 2, right panel). In contrast, the other indices (adjusted Ball and Hall, adjusted C, and adjusted determinant ratio) were not significantly different (Figure S3).

### 3.3. Infiltrating Immune Cells with EVI2B mRNA Level

**Figure 3:**
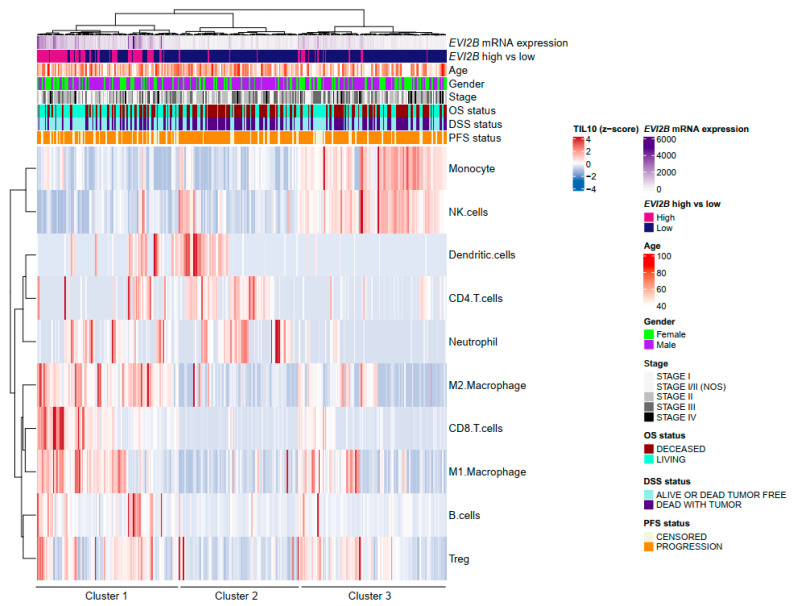
Heatmap of estimated TIL fractions by quanTIseq estimation [17] and clinical data showing three clusters in metastatic melanoma patients by non-hierarchical clustering.

To characterize the TIL population in the TME, we investigated the estimated fractions of TILs using the quanTIseq method [17]. Most of the *EVI2B* high tumors belonged to Cluster 1, and Cluster 2 or 3 consisted of *EVI2B* low tumors. Among *EVI2B* low tumors, Cluster 3 was enriched with particularly monocytes and NK cells, whereas Cluster 2 had dendritic cells, CD4+ T cells, and neutrophils. Notable, Cluster 1 (mostly *EVI2B* high tumor) had enriched cell fractions of CD8+ T cells, M1 and M2 macrophages, Treg, and B cells, while the other clusters (mostly *EVI2B* low tumor) had none of them (Figure 3). Similarly, the estimated TIL fraction by CIBERSORTx [29] showed one cluster in which most of patients were *EVI2B* high tumors, and had enriched CD8+ cells and M1 macrophages (Figure S4).

**Figure 4:**
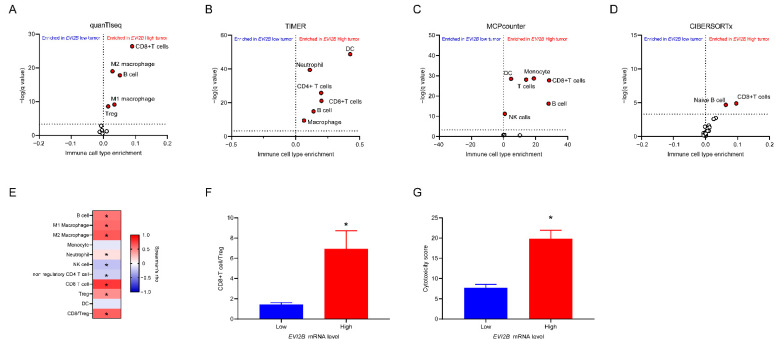
Volcano plots of the estimated TIL fraction in *EVI2B* high vs. *EVI2B* low patients: (**A**) quanTIseq; (**B**) TIMER; (**C**) MCPcounter; (**D**) CIBERSORTx. The cell fractions with Q value < 0.05 were considered to be significantly different fractions. (**E**) Spearman correlation of *EVI2B* gene expression with the TIL fractions estimated by the quanTIseq method, * *p* < 0.05. (**F**–**G**) Comparison of the CD8+ T cell/Treg ratio (**F**) and cytotoxicity score predicted by the MCPcounter method (**G**) between *EVI2B* high vs. *EVI2B* low patients. * *p* < 0.05 by Mann–Whitney Tests.

Next, we compared each estimated TIL population between the *EVI2B* high and *EVI2B* low patients. By the quanTIseq estimation [17], CD8+ T cells, M1 and M2 macrophages, B cells, and regulatory T cells (Treg) were significantly increased in *EVI2B* high patients (Figure 4A). Similarly, the other estimations by TIMER [27], MCPcounter [28], and CIBERSORTx [29] supported the increased CD8+ T cells and B cells (Figure 4B–D). Additionally, the *EVI2B* mRNA level was significantly correlated with the fractions of CD8+ T cells, Treg, M1/M2 macrophages, and B cells (Figure 4E). In melanoma patients, a balance of CD8+ T cells over Treg is essential for the prognosis. Here, we found that the CD8/Treg ratio and cytotoxicity score (by the MCPcounter method [28]) were significantly higher in *EVI2B* high patients (Figure 4F,G).

**Figure 5:**
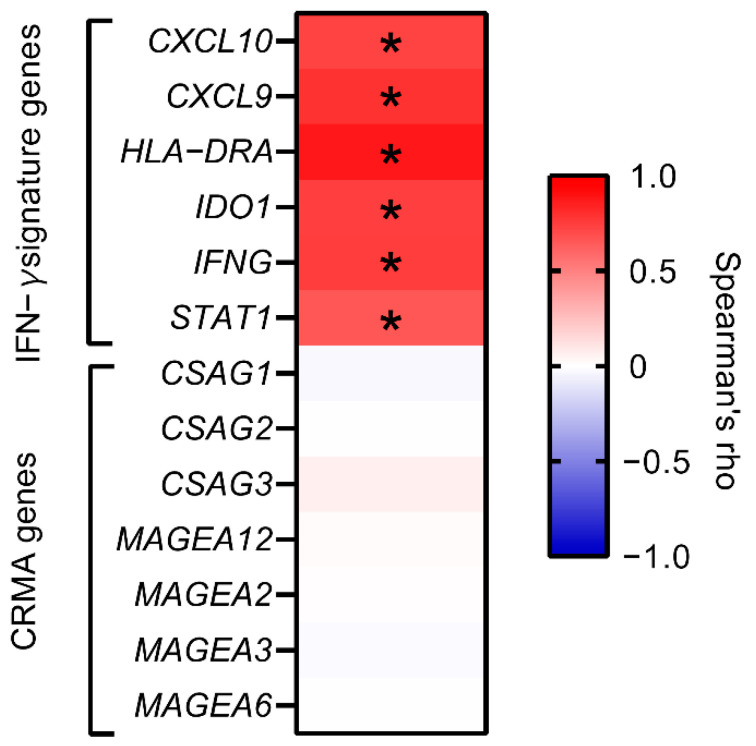
Spearman correlation of *EVI2B* gene expression with the IFN-γ related six genes (*IDO1*, *CXCL10*, *CXCL9*, *HLA-DRA*, *STAT1*, and *IFNG*) and the CRMA gene signature (*CSAG1*, *CSAG2*, *CSAG3*, *MAGEA2*, *MAGEA3*, *MAGEA6*, and *MAGEA12.* * *p* < 0.05.

IFN-γ is a critical driver of programmed death ligand-1 (PD-L1) expression in cancer and host cells, and the IFN-γ related gene signature can predict the response to the anti-PD-1 blockade (pembrolizumab) [18]. We evaluated the correlation of *EVI2B* gene expression in metastatic melanoma samples with the IFN-γ related gene signature (*CXCL10*, *CXCL9*, *HLA-DRA*, *IDO1*, *IFNG*, and *STAT1*) [18]. Interestingly, *EVI2B* gene expression positively correlated with all of the six genes (Figure 5). The CRMA gene signature (*CSAG1*, *CSAG2*, *CSAG3*, *MAGEA2*, *MAGEA3*, *MAGEA6*, and *MAGEA12*) is a predictor of resistance against anti-CTLA-4 blockade therapy [19]. Contrary to the IFN-γ related gene signature, we found no significant correlation with the CRMA gene signature (Figure 5 left). To further explore the association of *EVI2B* with immune-related genes, we evaluated the correlation with two sets of genes (immunostimulators and immunoinhibitors) [32] (Figure S5). Most of genes were positively correlated with *EVI2B* mRNA levels. Among the immunostimulators, only *PVR* and *CD276* were negatively correlated with *EVI2B*. VTCN1 was the only immunoinhibitory gene that was negatively correlated with *EVI2B*.

## 4. Discussion

Here, we report that *EVI2B* mRNA expression in tumor tissue was a favorable prognostic factor in metastatic melanoma patients. Melanoma tumors with higher *EVI2B* mRNA levels were characterized by a brisk pattern of TIL distribution with increased infiltration of multiple TILs, including CD8+ T cells. In *EVI2B* high patients, the ratio of CD8+ T cell to Treg was higher with increased cytotoxic activity. Moreover, *EVI2B* gene expression was positively correlated with IFN-γ related gene signature.

*EVI2B* (CD361 [7]) is a protein-coding gene that has been described as a common viral integration site in retrovirally-induced murine leukemia [8]. Granulocytic differentiation and functionality of hematopoietic progenitors are associated [9]. EVI2B works as a lymphocyte-specific marker in peripheral blood. *EVI2B* is one of the top 100 genes related to lymphocyte-specific attractor metagenes [10]. There are few publications on the association of *EVI2B* with cancer prognosis. In colorectal cancer, one study showed that *EVI2B* was one of the hub gene sets associated with tumor progression [11].

The current study adds a potential explanation for a favorable role of *EVI2B* in metastatic melanoma and shows that a higher *EVI2B* gene expression was associated with both increased TILs and favorable spatial arrangement of TILs. As compared with a non-brisk pattern of TILs, the brisk distribution pattern was associated with a high proportion of CD8+ T cells across tumors [12]. Consistent with this, we observed a higher CD8+ T cell fraction in the *EVI2B* high patient group. Moreover, the *EVI2B* high patients could have increased antitumor activity by a higher CD8+ T cell/Treg ratio, increased cytotoxicity score, and a positive correlation with IFN-γ and its related genes. Production of IFN-γ is observed by macrophages or certain types of lymphocytes, including effector CD8+ T cells, Th1 cells, NK, and NK T cells [33,34]. IFN-γ promotes increased tumor immunogenicity, cytotoxicity of CD8+ T cells, and migration of CD8+ T cells, NK cells, and NK T cells to a tumor site [35]. Therefore, IFN-γ in *EVI2B* high tumors mediates increased CD8+ T cells, contributing to a better prognosis. Of note, we observed some EVI2B low patients (i.e., Cluster 3) that harbored NK cells in tumor tissue (Figure 3). NK cells infiltration in melanoma can be a favorable prognostic factor [36]. NK cells in cancer tissue work for the recruitment/maturation of dendritic cells and Th1 differentiation [37]. However, our data showed that the NK cell-enriched patients lacked dendritic cells or CD8+ T cells (Figure 3). This could explain that enriched NK cells in EVI2B low patients are unlikely to contribute to a better prognosis.

The biomarker of a clinical response to an immune checkpoint blockade has been rigorously studied due to its limited response [38]. As for anti-PD-1 blockade, the IFN-γ gene signature (*IDO1*, *CXCL10*, *CXCL9*, *HLA-DRA*, *STAT1*, and *IFNG*) predicts the response to anti-PD-1 therapy in multiple tumors, including melanoma [18]. Recently, de Assis et al. showed that *ARNTL* (as known as *BMAL1*) was potentially a new clinical biomarker for T-cell based immunotherapy [39]. In the TCGA dataset, we found that *EVI2B* had a positive correlation with *ARNTL* (Figure S6). Moreover, EVI2B is highly correlated with immunostimulators and immunoinhibitors. Our data showed that *EVI2B* is closely associated with multiple sets of TILs and immunomodulatory genes; therefore, in this new era of immune oncology (IO), *EVI2B* could be a potentially interesting target for IO research in melanoma. The current study has multiple limitations. First, the publicly available cohorts that we used had a diverse background of patients with different disease stages or treatments, leading to difficulty in interpreting the results. Second, the results should be carefully interpreted because any random single or sets of genes can be significantly associated with a prognosis [40]. Third, the different tools or algorithms can harbor a potential bias due to different data processing or normalization. Further large studies would be necessary to demonstrate the predictive role of *EVI2B* mRNA level on the response to immune checkpoint blockade.

## 5. Conclusions

This study identifies the *EVI2B* mRNA expression as a prognostic biomarker in metastatic melanoma with IFN-γ related immune infiltration.

## Figures and Tables

**Table 1:** Multivariate Cox regression analysis on overall survival on TCGA dataset.

Variables	*EVI2B* Low	*EVI2B* High	*p* Value *
No. of patients (%)	272 (75%)	91 (25%)	
Age (years) (median, range)	57 (15–87)	54 (18–86)	0.491
Sex			0.211
Male	176	52	
Female	96	39	
Race			0.169
White	253	86	
Others (Asian or African American)	3	3	
NA	16	2	
TNM stage			0.375
Stage 0	6	1	
Stage I	55	20	
Stage I/II (NOS)	10	3	
Stage II	60	12	
Stage III	101	40	
Stage IV	18	3	
NA	22	12	

TCGA, The Cancer Genome Atlas; SKCM, skin cutaneous melanoma; NOS. not otherwise specified; NA, not available. * Category variables were compared by Fisher exact test or chi-square test and the continuous variables were compared by Mann–Whitney test.

**Table 2:** Multivariate Cox regression analysis on overall survival on the TCGA dataset.

Variable	HR	95% CI	*p*-Value
Age at diagnosis	1.021	1.010–1.032	<0.0001
Male (ref: Female)	0.885	0.629–1.245	0.483
Stage at initial diagnosis (ref: Stage 0/I)			
II	0.998	0.633–1.574	0.993
III	1.594	1.056–2.404	0.026
IV	2.967	1.355–6.498	0.007
Tumor purity	0.777	0.294–2.056	0.611
*EVI2B* mRNA level	0.716	0.610–0.840	<0.0001

*N* = 368. TCGA, The Cancer Genome Atlas; HR, hazard ratio; CI, confidence interval; ref, reference.

## Data Availability

All the data used in this study are publicly available.

## Supplementary Materials

The following are available online at https://www.mdpi.com/article/10.3390/cancers13164110/s1, Table S1: Multivariate Cox regression analysis with dichotomized *EVI2B* mRNA levels on overall survival of the TCGA dataset, Figure S1: Distribution of *EVI2B* mRNA levels in metastatic melanoma of the TCGA cohort, Figure S2: Kaplan–Meier survival curves in *EVI2B* high vs. *EVI2B* low patients with a 50% cut-off value in metastatic melanoma patients of the TCGA, GSE65904, and GSE19234 datasets, Figure S3: Distribution of *EVI2B* mRNA levels in metastatic melanoma of the TCGA cohort, Figure S4: Heatmap of estimated TILs fractions by CIBERSORTx and clinical data showing two clusters in metastatic melanoma patients by non-hierarchical clustering, Figure S5: Spearman correlation of *EVI2B* mRNA level with immunostimulators and immunoinhibitors, Figure S6: Spearman correlation of *EVI2B* mRNA level with *ARNTL* (*BMAL1*) in metastatic melanoma patients of the TCGA cohort. TPM, transcripts per million.
