# Supplementary material for: EVI2B Is a New Prognostic Biomarker in Metastatic Melanoma with IFNgamma Associated Immune Infiltration
Source: Cancers (Basel). 2021 Aug 15;13(16):4110. doi: 10.3390/cancers13164110 (PMC8391972; doi:10.3390/cancers13164110)
Supplement: Supplementary file 1 [file cancers-13-04110-s001.zip › cancers-1317238-supplementary.pdf]

Supplementary Material: *EVI2B* Is a New Prognostic Biomarker in Metastatic Melanoma with IFNgamma Associated Immune Infiltration

Satoru Yonekura and Kosuke Ueda

Table S1. Multivariate Cox regression analysis with dichotomized *EVI2B* mRNA levels on overall survival of the TCGA dataset.

Variable	HR	95% CI	*p*-Value
Age at diagnosis	1.020	1.010-1.032	0.0001
Male (ref: Female)	1.069	0.768-1.489	0.8040
Stage at initial diagnosis (ref: Stage 0/I)			
II	1.181	0.760-1.835	0.4602
III	1.831	1.229-2.728	0.0030
IV	3.167	1.452-6.908	0.0038
EVI2B high (ref : low)	0.5022	0.329-0.767	0.0014

TCGA: The Cancer Genome Atlas; HR, hazard ratio; CI, confidence interval; ref, reference.

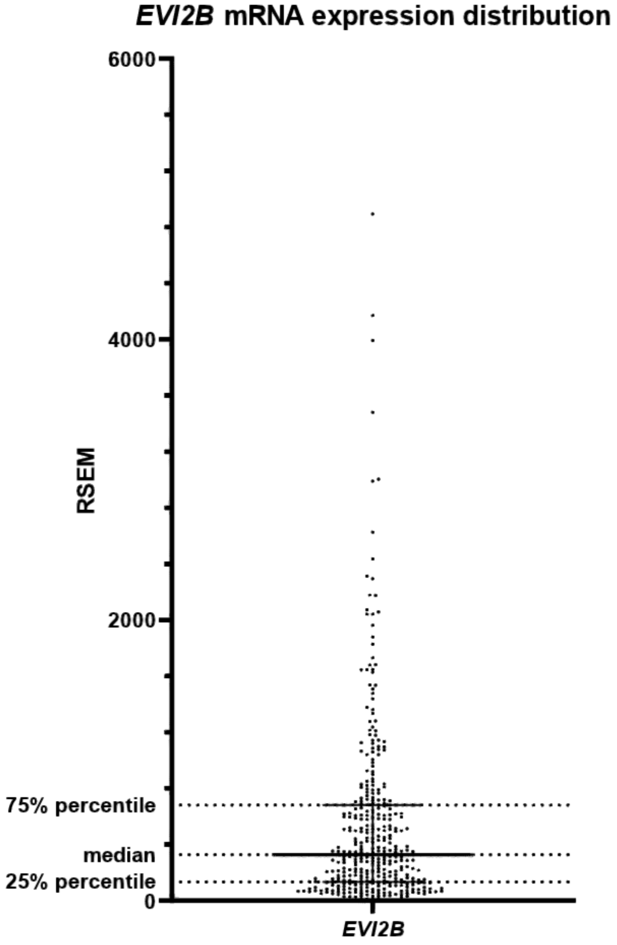

Figure S1. Distribution of *EVI2B* mRNA levels in metastatic melanoma of the TCGA cohort. RSEM, RNA-Seq by Expectation-Maximization.

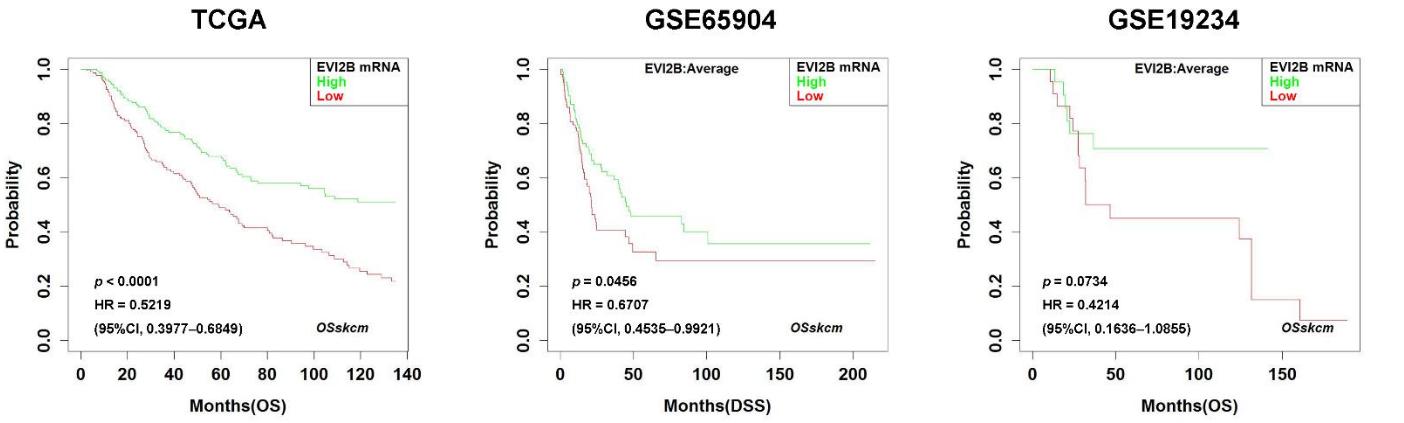

Figure S2. Kaplan–Meier survival curves in *EVI2B* high vs. low patients with a 50% cut-off value in metastatic melanoma patients of the TCGA, GSE65904, and GSE19234 datasets. The outcomes are overall survival (OS, **left** and **right panels**) and disease-free survival (DSS, **middle panel**).

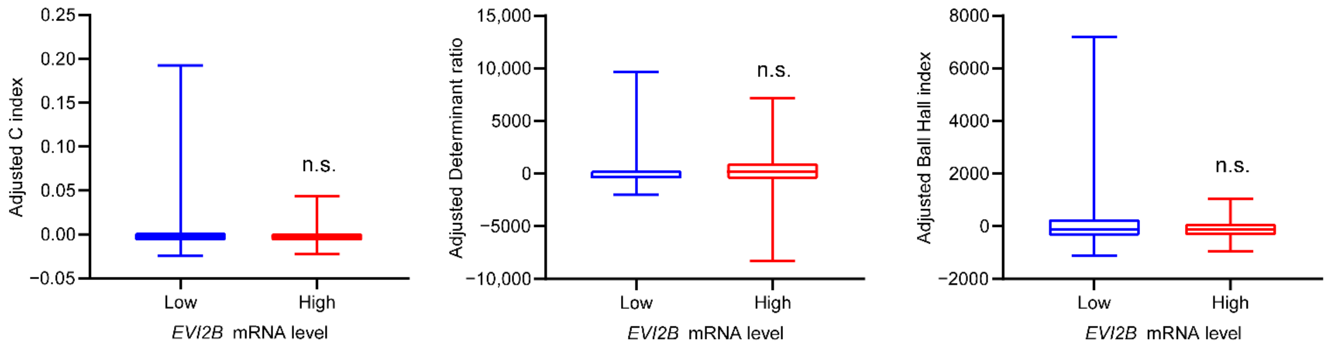

Figure S3. Distribution of *EVI2B* mRNA levels in metastatic melanoma of the TCGA cohort. RSEM, RNA-Seq by Expectation-Maximization. n.s., not significant.

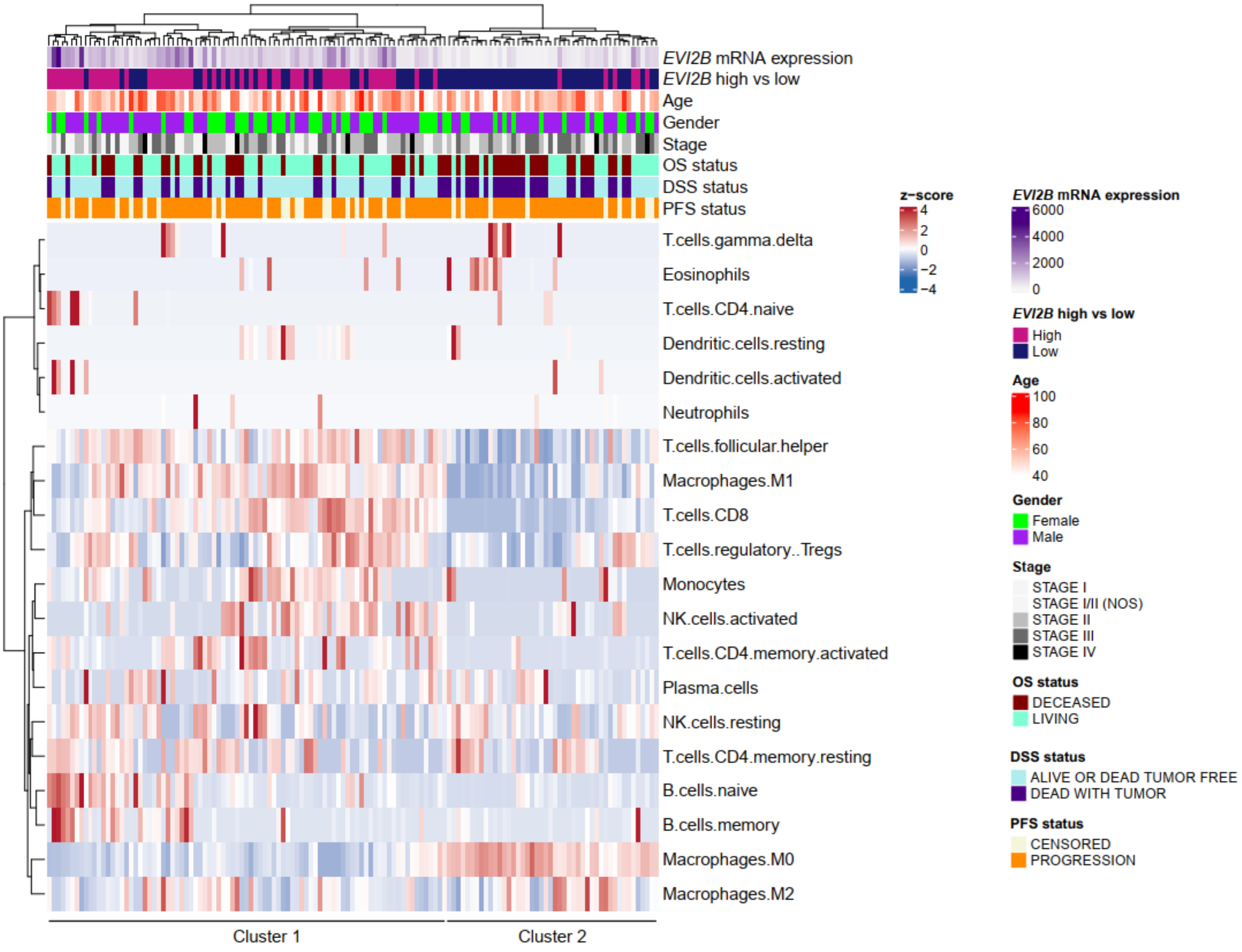

Figure S4. Heatmap of estimated TILs fractions by CIBERSORTx [29] and clinical data showing two clusters in metastatic melanoma patients by non-hierarchical clustering.

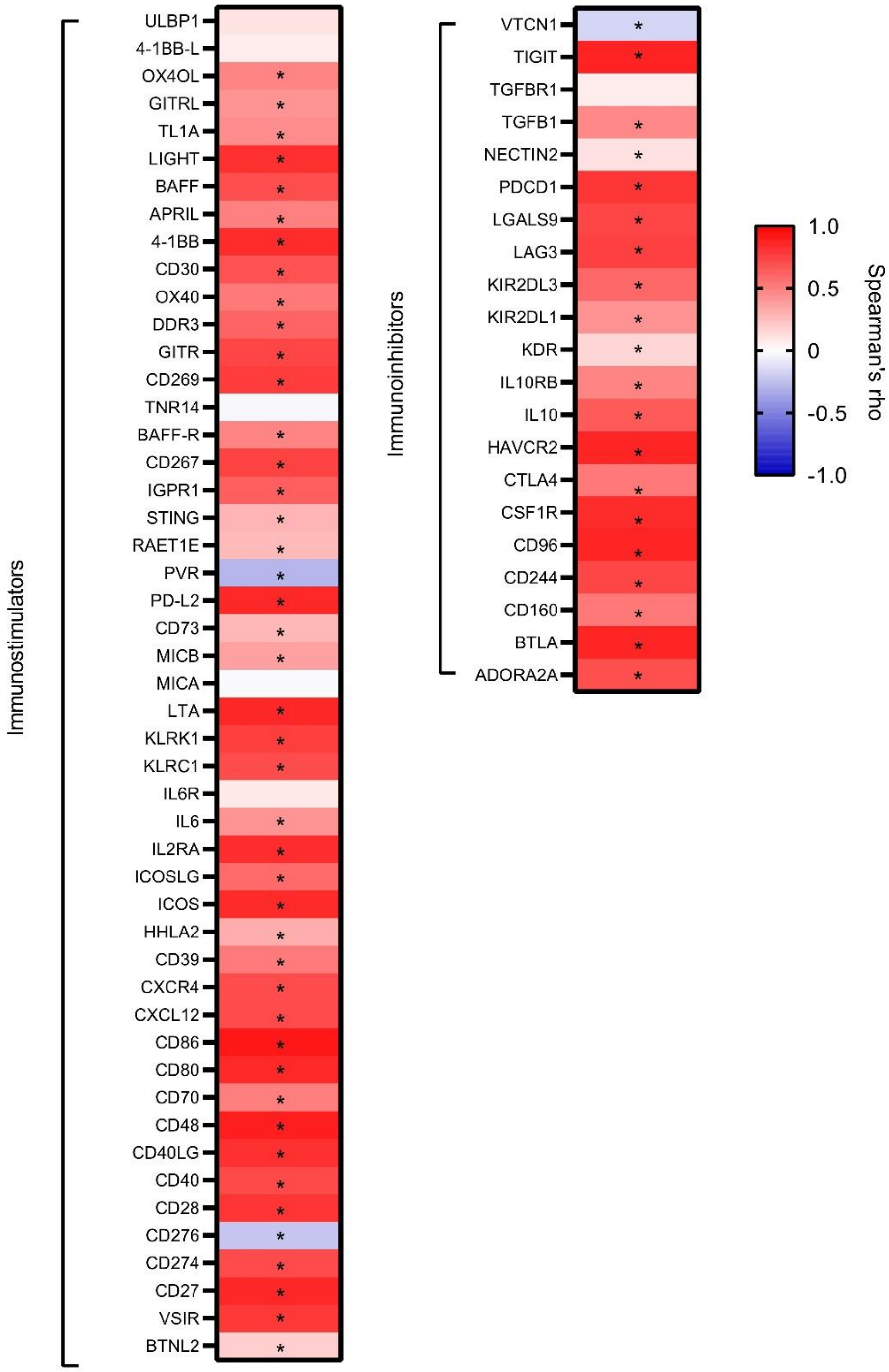

Figure S5. Spearman correlation of *EVI2B* mRNA level with immunostimulators and immunoinhibitors [32].

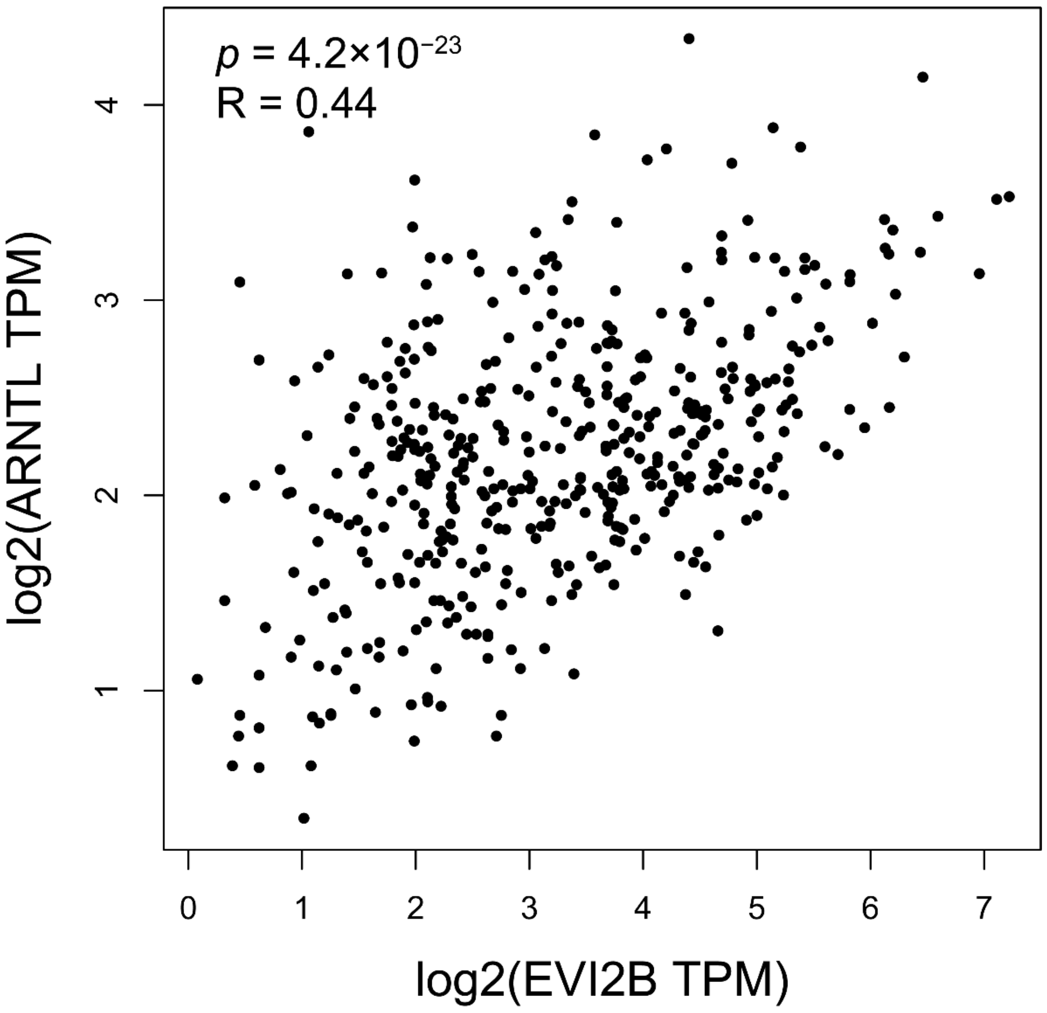

Figure S6. Spearman correlation of *EVI2B* mRNA level with *ARNTL* (*BMAL1*) in metastatic melanoma patients of the TCGA cohort. TPM, transcripts per million.
